# Country perspectives on integrated approaches to maternal and child health: the need for alignment and coordination

**DOI:** 10.2471/BLT.15.168823

**Published:** 2016-05-02

**Authors:** Pascal Bijleveld, Blerta Maliqi, Paul Pronyk, Jennifer Franz-Vasdeki, Bennett Nemser, Diana Sera, Renee van de Weerdt, Benedicte Walter

**Affiliations:** a Reproductive, Maternal and Newborn Health Strategy and Coordination Team, United Nations Children’s Fund, New York, United States of America (USA).; b Department of Maternal, Newborn, Child and Adolescent Health, World Health Organization, avenue Appia 20, 1211 Geneva 27, Switzerland.; c United Nations Children’s Fund, New York, USA.; d Partnership for Maternal, Newborn and Child Health, World Health Organization, Geneva, Switzerland.; e United Nations Population Fund, New York, USA.

The sustainable development goals (SDGs) and updated *Global strategy for women’s, children’s and adolescents’ health (2016–2030) *call upon countries and the global community to transform health systems and society.^1^^,^^2^ Additional investments in women’s, children’s and adolescents’ health are essential to achieve the ambitious agenda that has been set. Support for nationally-identified reproductive, maternal, newborn and child health priorities needs to be better coordinated and new mechanisms, such as the Global Financing Facility, need to be used to expand domestic financing and attract additional resources.^3^^,^^4^ The relevant global frameworks have to be translated into actions at the country level and, for this, the lessons learnt from previous engagement with individual countries may give useful guidance.

The Reproductive, Maternal, Newborn and Child Health Fund was established in 2013 to support countries with the highest burden of preventable maternal and child deaths and help direct such countries towards millennium development goals (MDGs) 4 and 5 (to reduce child mortality and to improve maternal health by 2015). The fund’s main aims were to provide catalytic resources to fill the critical gaps identified by countries and facilitate greater integration of financing and intervention programmes. At the time of the fund’s launch, integration was complicated by the proliferation of multiple new initiatives under the Every Woman Every Child umbrella – e.g. A Promised Renewed, Family Planning 2020 and the United Nations (UN) Commission on Life-Saving Commodities, etc.^5^

In 2013, under the stewardship of heath ministries and with the support of the H4+ partnership, UN agencies and partners, a country engagement process was outlined to identify implementation gaps across the reproductive, maternal, newborn and child health spectrum. The objectives were to improve the alignment and integration of partners and programmes and to provide catalytic support for prioritized gaps. Using the latest relevant national and subnational data, a situation analysis was conducted to evaluate progress and identify key bottlenecks. Demand for reproductive, maternal, newborn and child health was then evaluated so that the interventions and strategies needed to achieve MDGs 4 and 5 could be determined. In many cases, the priorities identified had already appeared in existing national plans. In-country dialogues with key stakeholders then identified opportunities to strengthen national coordination and alignment. Forward-looking resource mapping was used to assess the financial resources that were – or might become – available to support reproductive, maternal, newborn and child health from domestic and external sources. Finally, prioritized gaps were identified against which further resource alignment could take place and new funding – e.g. from the Reproductive, Maternal, Newborn and Child Health Fund – mobilized.

The country perspectives that we present in this article (Table 1) come from the health ministries, civil society organizations and partner agencies from 16 countries in sub-Saharan Africa that collectively received over 180 million United States dollars from the Reproductive, Maternal, Newborn and Child Health Fund (Box 1 and Box 2). These perspectives were generated from a synthesis of facilitated group discussions that took place, among representatives from all 16 focus countries, during a week-long workshop in October 2015.^6^

**Table 1:** Challenges and potential solutions for more effective planning and implementation of national reproductive, maternal, newborn and child health strategies, 2016

Challenge identified	Details	Potential solution
Lack of an enabling environment	The RMNCH landscape is complex, with multiple parallel global initiatives.	While global initiatives provide important political momentum and technical focus, some consolidation would be beneficial. Coordinated application at the country-level is essential.
Multiple RMNCH coordinating mechanisms	There is fragmentation and duplication at country level.	To provide coherence and alignment to financing and programming, ministry-led coordination platforms that bring all partners – including civil society and the private sector – together and work across the RMNCH spectrum should be supported.
Lack of clarity on available resources	There is an absence of transparency in forward-looking domestic and partner contributions. This makes planning and budgeting difficult.	Greater transparency in resource commitments is essential. Consolidation of external financing channels can increase stability and allow for more effective planning.
Support for planning and implementation	Additional support is required for the translation of global learning and best practice to planning and implementation at country level.	Greater investment in local building of management capacity, more streamlining, greater coherence and quality control for regionally or globally developed tools and more flexible and responsive backstopping, at regional and global levels, to allow immediate national technical needs and demands to be met.
Managing competing priorities	In the face of limited resources, balancing competing programmatic interests with longer-term national priorities can be difficult.	Nationally-driven consolidation of domestic and partner RMNCH resources can reduce fragmented planning and help address longer-term priorities. Evidence-informed approaches to national and subnational planning are critical. Civil society has an important role to play.
Flexible longer-term commitments	Short-term vertical commitments and administrative hurdles from partners adversely affect planning and implementation.	Central need for the consolidation of resources, coordination of programming and support for longer-term horizons that align with national planning cycles.
Integrated platforms for monitoring and accountability	Efforts to track progress at the population and facility level are fragmented, periodic reporting systems have been of poor quality and not timely and information flow has been in one direction – i.e. towards national managers.	Greater harmonization and integration of data collection tools and platforms are essential. Better application of innovations in real-time reporting can support timely and bidirectional information flow and improve accountability and action.

RMNCH: reproductive, maternal, newborn and child health.

Box 1 **Kenya – partner coordination in six high-burden counties**

Kenya received 14.9 million United States dollars from the Reproductive, Maternal, Newborn and Child Health Fund to contribute to the reduction of maternal and newborn mortality in the counties of Isiolo, Lamu, Mandera, Marsabit, Migori and Wajir. Together, despite holding just 10% of the country’s population, these six counties contribute close to 50% of the country’s maternal deaths (UNFPA Kenya Country Office, unpublished data, December 2014). The targeted support was in line with national priorities to enact transformational change through decentralization of health sector management. The ministry-led country engagement process brought together a range of partners including the United Nations Children’s Fund, the United Nations Population Fund (UNFPA), the World Health Organization and various governmental and nongovernmental stakeholders – particularly the relevant county health management teams, the African Medical Research Foundation, the Kenya Red Cross and the Liverpool School of Tropical Medicine.

The engagement process resulted in the crowding-in of additional resources from various sources, including local county funds, new development partners – e.g. the Danish International Development Agency and the Swedish International Development Cooperation Agency – and, most recently, the Global Finance Facility. A new public–private partnership was launched in Kenya, in support of the updated global strategy and under the umbrella of the First Lady’s Beyond Zero Campaign, with the aim of reaching over 3.5 million women, neonates, children, adolescents and family members in the six high-burden counties by 2020. Companies that have signed onto the initiative include GlaxoSmithKline, Huawei, Merck Sharp & Dohme, Philips and Safaricom. The Kenya Healthcare Federation and the UNFPA will convene partners and coordinate the implementation of this joint commitment. The partnership will target several activities including: strengthening supply-chain management for health commodities; increasing availability and demand for youth-friendly health services; capacity building for health professionals; innovations in health management systems; increasing access to energy for facilities; youth empowerment; research; and resource mobilization.

Box 2 **Malawi – resource mapping to address inequities**

Malawi is a recipient of 11.5 million United States dollars from the Reproductive, Maternal, Newborn and Child Health Fund, to be used to ensure the availability, equitable access and rational use of high-quality safe and efficacious medicines at an affordable cost and accelerate the reduction of maternal and neonatal morbidity and mortality.

In a bid to reduce duplication and fragmentation in financing and programming, Malawi underwent a country engagement process. The aim was to ensure that the additional short-term catalytic financing from the fund was responding to existing plans and strategies, complementary to other financing streams and prioritized to achieve maximum impact. An important element of an evidence-informed process of priority setting was the mapping of existing and committed financial resources for reproductive, maternal, newborn and child health that were available for the country.

Annual resource mapping in Malawi, which captures all resources planned for the health sector, proved an invaluable asset in generating evidence to inform resource allocation and to strengthen national ownership and coordination of the reproductive, maternal, newborn and child health response among the government and partners. The mapping highlighted district inequities in resources and programming, extreme fragmentation among partners and inefficiencies in resource allocations. The mapping data were used to fine tune geographical prioritization towards greater equity and ensure that resources were targeted towards areas where they were needed most.

By 2016, a range of lessons had been learnt from this country engagement process. There was a lack of enabling environment. At country level, there was persistent confusion around the multitude of global and regional initiatives on reproductive, maternal, newborn and child health. This resulted in high transaction costs for ministries and duplication and fragmentation in financing and programming. Although there was a compelling global discourse and commitment to improving alignment, this was not always mirrored by actions on the ground.

The multiple coordinating mechanisms also caused problems. Despite the promotion of a single established coordination mechanism, the focus countries acknowledged that multiple platforms led by the health ministries but involving a wide range of stakeholders often existed and were often needed. These platforms had different levels of authority, had focus on the political coordination or technical expertise and had inputs from various sectors and stakeholders. Typically, the reproductive, maternal, newborn and child health platform formed a subset of a larger health platform and operated independently, with insufficient civil society engagement. Government stewardship and the alignment of the planning process for the related activities and partners with the broader national planning cycles facilitated greater coordination. Integrated financing and programming across the spectrum of reproductive, maternal, newborn and child health activities enabled more effective planning and more operational synergies.

Focus countries also reported a lack of clarity on resources at the country level. Alignment of financing at country level required dedicated efforts from ministries and partners. All stakeholders recognized the importance of transparency. However, the focus countries faced challenges in getting stakeholders to deliver information on the future provision of financial resources and suggested that, in some cases, a legal framework might help to formalize the transparency they needed.

Timely technical support and knowledge of existing tools and planning processes were essential to ensure that country needs were met efficiently and rapidly. Countries valued additional facilitation and support – both local and external – to assist in the analysis, prioritization and costing of plans. Streamlining advice and input from providers such as the Global Fund to Fight Aids, Tuberculosis and Malaria, the UN and the United States Agency for International Development was important to success. More must be done to build long-term national capacity. Finally, as the reproductive, maternal, newborn and child health space is dynamic, mechanisms to ensure that global learning can inform practice at the country level during the planning and implementation cycles are critical.

Given few resources, countries faced difficult choices around which strategies to prioritize. Greater integration of reproductive, maternal, newborn and child health financing – rather than more fragmented disease- or priority-specific approaches – facilitated more robust prioritization. In addition, an evidence-informed and transparent approach, particularly with the involvement of civil society, helped ensure that resources got where they were needed most.

Focus countries voiced a range of perspectives regarding how to optimize implementation. The use of established systems and partners for planning, financing and implementation helped improve efficiencies. There were often major administrative hurdles in the countries’ dealings with key external development partners and these created unnecessary delays. Greater administrative harmonization is needed. Flexibility in how resources could be deployed allowed any immediate needs to be addressed and a rapid response to any time-sensitive bottlenecks. A financing horizon that was longer than the typical cycle of 12–24 months was felt to be important in facilitating longer-term planning and enhancing the consistency of the networks required for effective implementation.

Since 2013, various tools and systems have been introduced and/or strengthened by the focus countries to facilitate the real-time tracking of reproductive, maternal, newborn and child health indicators. These include scorecards, commodity-dashboards, text-message-based stock-out tracking and district health information systems. Focus countries voiced strong support for the integration of various platforms into a common monitoring system – which could facilitate the timely review of key metrics and help achieve targets and coordinate investments – while recognizing that information gaps, poor data quality and poor interoperability remain as challenges. Additional attention should be given to ensuring that monitoring data do not flow just towards national managers but also reach providers and decision-makers at facility and community level. Finally, the application of monitoring tools to improve performance management systems – e.g. results-based financing – should be expanded.

In summary, the MDGs have come to a close and the updated global strategy and SDGs take the reproductive, maternal, newborn and child health agenda forward. Success will depend on the ability and willingness of countries and the global community to leverage joint efforts and capitalize on existing knowledge, resources and funding. Country perspectives from recent engagement through the Reproductive, Maternal, Newborn and Child Health Fund have generated important lessons to inform this agenda.

While the various global initiatives on reproductive, maternal, newborn and child health have generated technical and political momentum in countries around several previously neglected priorities, better coordination of the initiatives’ application on the ground is essential. Existing ministry-led coordination platforms that draw together partners, civil society and the private sector are important in enhancing integration and reducing the burden on ministries that are already over-stretched. Greater transparency in forward-looking resource commitments and the consolidation of external financing channels would facilitate the development of planning synergies. If planning and implementation are to be effective and efficient, there needs to be global agreement on a common suite of best-practice tools and resources for reproductive, maternal, newborn and child health. To strengthen national capacity further, a so-called one-stop shop for key resources, tools, training and support might be created. There is a need for longer-term and more integrated financing and programming arrangements that align with national planning cycles. Finally, a focus on results is central to operationalizing the global strategy. Country-led efforts to enhance accountability and action can deepen the gains made in reproductive, maternal, newborn and child health and strengthen health system resilience in the long term.
